# De novo substitutions of *TRPM3* cause intellectual disability and epilepsy

**DOI:** 10.1038/s41431-019-0462-x

**Published:** 2019-07-05

**Authors:** David A. Dyment, Paulien A. Terhal, Cecilie F. Rustad, Kristian Tveten, Christopher Griffith, Parul Jayakar, Marwan Shinawi, Sara Ellingwood, Rosemarie Smith, Koen van Gassen, Kirsty McWalter, A. Micheil Innes, Matthew A. Lines

**Affiliations:** 1 Children’s Hospital of Eastern Ontario Research Institute, University of Ottawa, Ottawa, ON Canada; 2 Department of Pediatrics, University of Ottawa, Ottawa, ON Canada; 3 Department of Genetics, University Medical Centre Utrecht, Utrecht, the Netherlands; 4 Department of Medical Genetics, Oslo University Hospital, Oslo, Norway; 5 Department of Medical Genetics, Telemark Hospital Trust, Skien, Norway; 6 University of South Florida, Tampa, FL USA; 7 Nicklaus Children’s Hospital, Miami, FL USA; 8 Department of Pediatrics, Division of Genetics and Genomic Medicine, Washington University School of Medicine, St. Louis, MO USA; 9 Department of Pediatrics, Division of Genetics, Maine Medical Center, Portland, ME USA; 10 GeneDx, Gaithersburg, MD USA; 11 Department of Medical Genetics and Alberta Children’s Hospital Research Institute, Cumming School of Medicine, University of Calgary, Calgary, AB Canada

**Keywords:** Medical genetics, Epilepsy

## Abstract

The developmental and epileptic encephalopathies (DEE) are a heterogeneous group of chronic encephalopathies frequently associated with rare de novo nonsynonymous coding variants in neuronally expressed genes. Here, we describe eight probands with a DEE phenotype comprising intellectual disability, epilepsy, and hypotonia. Exome trio analysis showed de novo variants in *TRPM3*, encoding a brain-expressed transient receptor potential channel, in each. Seven probands were identically heterozygous for a recurrent substitution, p.(Val837Met), in TRPM3’s S4–S5 linker region, a conserved domain proposed to undergo conformational change during gated channel opening. The eighth individual was heterozygous for a proline substitution, p.(Pro937Gln), at the boundary between TRPM3’s flexible pore-forming loop and an adjacent alpha-helix. General-population truncating variants and microdeletions occur throughout *TRPM3*, suggesting a pathomechanism other than simple haploinsufficiency. We conclude that de novo variants in *TRPM3* are a cause of intellectual disability and epilepsy.

## Introduction

The developmental and epileptic encephalopathies (DEE) are a heterogeneous group of disorders characterized by epilepsy with comorbid intellectual disability (ID). Rare nonsynonymous coding variants in genes encoding ion channels, cell-surface receptors, and other neuronally expressed proteins are identifiable in one about quarter of affected individuals [1–3]. Most identified variants in individuals with DEE are in-frame, de novo, and recurrent across unrelated kindreds [2].

Transient receptor potential (TRP) channels are a superfamily of gated cation channels sensitive to a variety of physical and chemical stimuli [4]. Seven subfamilies are recognized [5]. TRP channels are implicated in several Mendelian disorders, including polycystic kidney disease (OMIM #613095), mucolipidosis type IV (#252650), amyotrophic lateral sclerosis–dementia–parkinsonism complex (#105500), and others [5]. All TRP proteins share a common six-transmembrane-helix architecture with fourfold radial symmetry, distinct voltage-sensing and pore-forming domains, and variable N- and C-terminal cytoplasmic tails [4]. Some TRP proteins mediate sensory stimuli, e.g., noxious heat (TRPV1, TRPM3, and TRPA1) and cold (TRPM8); others are receptor-operated, and/or responsive to cellular stimuli including osmolarity, intracellular calcium, and/or chemical ligands [4].

In this study, we present eight individuals with a neurodevelopmental phenotype comprising ID, hypotonia, epilepsy (seven individuals), and a recognizable craniofacial gestalt; exome sequencing showed de novo substitutions of a TRP (melastatin-related) channel, TRPM3, in each. Seven of eight probands were heterozygous for a recurrent substitution, NM_020952.4:c.2509G>A, NP_066003.3:p.(Val837Met), altering a conserved residue previously implicated in channel gating. We propose that de novo substitutions of *TRPM3* are a cause of ID and epilepsy.

## Materials and methods

All procedures were in accord with the Declaration of Helsinki. Following suitable informed consent, exome sequencing of each proband plus their unaffected parents was performed on an accredited clinical basis according to standard protocols. Cohort assembly was accomplished by distributed case-matching in GeneMatcher [6]. Clinical and genetic data were provided by individual physician coauthors in accordance with local research and ethics requirements. The variants are deposited in ClinVar with accession numbers SCV000891785 and SCV000891786.

## Results

### Clinical findings

**Fig. 1:**
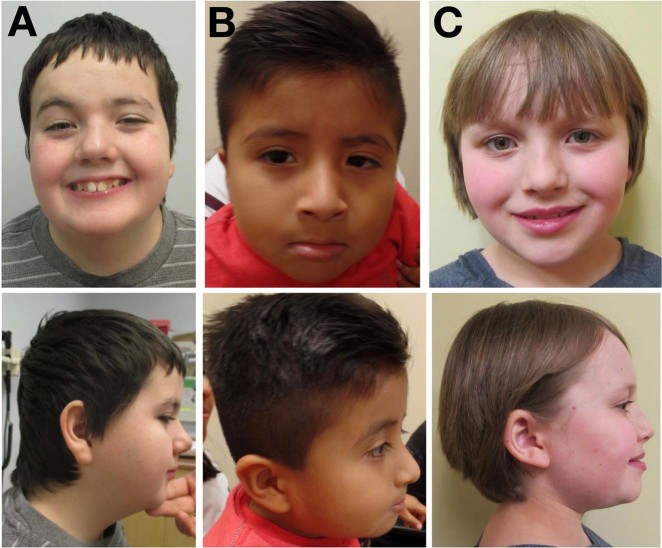
Craniofacial morphology. **a** Individual 1, (p.Val837Met), age 12.5 years. **b** Individual 2, (p.Val837Met), age 4.5 years. **c** Individual 8, (p.Pro937Gln), age 10.8 years

The probands are eight unrelated individuals with a symptom complex comprising moderate-to-severe global developmental delay (eight individuals), hypotonia or mixed tone abnormality (eight individuals), electrographically confirmed epilepsy (seven individuals), and/or variable minor anomalies (Table 1). Seizures corresponded to several clinical types (absence, generalized tonic-clonic, infantile spasms, and subclinical, including electrographic status epilepticus of sleep), were noted in infancy or early childhood, and were generally responsive to standard medical management. Electroencephalography showed nonspecific epileptiform activity. Brain MRI was normal in six individuals, and showed nonspecific volume loss in two individuals. Other associated anomalies, each observed in a minority of probands, included: Strabismus (four individuals), scoliosis (three individuals), talipes equinovarus (two individuals), athetoid movements in infancy (two individuals), C1 vertebral anomalies (two individuals), pectus excavatum (one individual), cryptorchidism (one individual), micropenis (one individual), and hip dysplasia (one individual). There was no consistent growth phenotype. The craniofacial gestalt was nondysmorphic, although shared features included a broad forehead, prominent nasal root, bulbous nasal tip, short philtrum, micrognathia, and prominent lobule of the ear (Fig. 1). One individual was described to have a heightened threshold for pain; a second individual had a history of repeated handwashing with scalding water. In no case was altered heat or pain sensitivity the primary reason for referral.

**Table 1:** Clinical and molecular characteristics

Individual	1	2	3	4	5	6	7	8
*TRPM3* variant								
cDNA (NM_020952.4)	c.2509G>A	c.2509G>A	c.2509G>A	c.2509G>A	c.2509G>A	c.2509G>A	c.2509G>A	c.2810C>A
Polypeptide (NP_066003.3)	p.(Val837Met)	p.(Val837Met)	p.(Val837Met)	p.(Val837Met)	p.(Val837Met)	p.(Val837Met)	p.(Val837Met)	p.(Pro937Gln)
Genomic DNA (NC_000009.11)	g.73213379C>T	g.73213379C>T	g.73213379C>T	g.73213379C>T	g.73213379C>T	g.73213379C>T	g.73213379C>T	g.73168145G>T
Zygosity	Heterozygous	Heterozygous	Heterozygous	Heterozygous	Heterozygous	Heterozygous	Heterozygous	Heterozygous
Segregation	De novo	De novo	De novo	De novo	De novo	De novo	De novo	De novo
Clinical features								
Gestation (weeks)	38	40	42	39	38 + 3	40	39	Term
Perinatal history	C/S	N	N	N	N	N	C/S	C/S (repeat)
Birth weight (kg)	NR	3.6	3.2	3.48	3.378	3.89	3.1	2.9
Sex	M	M	F	M	M	M	M	F
Age (years)	16	4.75	6	5.9	6.25	28	38	8.1
Height (cm)	164.5 (*Z* = −1.0)	105.1 (*Z* = −0.7)	110 (*Z* = −1.0)	117 (*Z* = + 0.3)	116 (*Z* = −0.3)	NR	169.5 (*Z* = −1.3)	115.7 (*Z* = −2.0)
Weight (kg)	73.3 (*Z* = + 1.0)	17.6 (*Z* = −0.1)	17.8 (*Z* = −0.9)	24.5 (*Z* = + 1.4)	22 (*Z* = + 0.3)	NR	63.2 (*Z* = −1.0)	27.8 (*Z* = + 0.6)
BMI (kg/m^2^)	27.1 (*Z* = + 1.8)	15.9 (*Z* = + 0.5)	14.7 (*Z* = −0.4)	17.9 (*Z* = + 1.7)	16.3 (*Z* = + 0.7)	NR	22.1 (*Z* = −0.0)	22.3 (*Z* = + 2.0)
OFC (cm)	55.8 (15 years, 8 months) (*Z* = + 0.2)	49.5 (*Z* = −0.8)	51 (*Z* = + 0.2)	55 (*Z* = + 2.1)	53.2 (*Z* = + 0.7)	56 (Z = 0)	57 (*Z* = + 0.5)	52 (*Z* = + 0.2)
Developmental delay/intellectual disability	+ (Severe)	+ (Moderate)	+ (Moderate-to-severe)	+ (Severe)	+ (Severe)	+ (Severe)	+ (Moderate)	+ (Moderate-to-severe)
Ambulate independently (age achieved)	+ (5 years)	+ (With walker) (3 years)	−	+ (With walker)	+ (4.5 years)	−	+ (4 years)	+ (3.5 years)
Any speech (age attained)	+ (5 years)	−	−	−	−	−	+ (5 years)	+ (2.5 years)
Combine words/signs	+	−	−	−	−	−	+ (Signs)	+ (Sentences)
Toilet independently (age attained)	+ (9 years)	−	−	−	−	−	NR	(4 years)
Autism-like features	+	NR	+	+	+	−	NR	−
Electrographically confirmed seizures	+	+	+	+	+	+	Unconfirmed	+
Seizure types	Absence	Infantile spasms	GTC	Subclinical, including ESES	NR	Absence and GTC	Absence	Absence
Age of first clinical seizure	Absence-like episodes in infancy; first documented EEG abnormalities at 7 years	NR	NR	EEG abnormalities at 3 years	11 months	9 months	<1 year	2 years
Current anticonvulsant therapy	Levetiracetam (initial); none (current)	NR	NR	Diazepam qHS (with improvement in ESES)	Levetiracetam		None	Lamotrigine
Age of last clinical seizure	NR (untreated follow-up EEG at age 15 was normal)	NR	NR	NR	5 years, 9 months	26 years (EEG remains pathological with diffuse high-amplitude activity)	NR	6 years
Hypotonia	+	+	+	+	+	−	+ (mixed tone abnormality)	+
Craniofacial gestalt	Nondysmorphic	Nondysmorphic	Nondysmorphic	NR	NR	NR	Distinctive	Nondysmorphic
Morphological features	Broad forehead, deeply set eyes, ptosis, bulbous nasal tip, micrognathia, prominent lobule of ear, tapering fingers	Short philtrum, long nose, turricephaly	NR	Broad forehead, deeply set eyes, flat midface, short philtrum, micrognathia, broad halluces, fifth-finger clinodactyly, pectus excavatum	Broad forehead, low nasal bridge, unilateral preauricular pit, short broad thumbs	Micrognathia, high palate	Mild facial asymmetry, ptosis, telecanthus, bulbous nasal tip, micrognathia, short neck, low hairline	Broad forehead, deeply set eyes, upslanting palpebral fissures, bulbous/upturned nasal tip, short philtrum, large oral aperture, facial capillary hemangioma
Other clinical features	C1 spinal stenosis; Chiari I malformation; scoliosis; torticollis; plagiocephaly; thickened filum terminale; bilateral talipes equinovarus; strabismus (exotropia OU)	EMG/NCS normal	−	Strabismus	Cryptorchidism, micropenis, bilateral talipes equinovarus	Neonatal hypoglycemia; unilateral hip dysplasia; scoliosis	Atlanto-occipital fusion, strabismus (exotropia), hands held ‘fisted’ until 9 months, athetoid movements in infancy, pes planus	Choreoathetoid movements in infancy (age 5 months), strabismus, scoliosis
Brain MRI	Possible mild cerebral volume loss	Normal	Normal	Normal	Ventriculomegaly, nonspecific periventricular white matter hyperintensities	Normal	Normal	Normal
Apparent heat or pain insensitivity	+ (Heat)	NR	NR	NR	+ (Pain)	−	NR	NR
Genetic investigations								
aCGH	Normal	Normal	Normal	Normal	Normal	Normal	Normal	Normal
Fragile X	Normal	Normal	Normal	Normal	N/A	Normal	Normal	N/A
Other (nondiagnostic) genetic investigations	ID panel (170 genes), *PHF6*	NR	*MECP2*, SMA	NR	NR	NR	9 gene XLID panel, *MCT8*	mtDNA, *POLG*, *DGUOK*, *TK2*, *SUCLA2*, ID panel (196 genes)

*aCGH* microarray-based comparative genomic hybridization, *DOL* day of life, *EMG* electromyograph, *ESES* electrographic status epilepticus of sleep, *GTC* generalized tonic-clonic, *ID* intellectual disability, *N* normal, *N/A* not applicable, *NCS* nerve conduction study, *NR* not recorded, *OFC* occipitofrontal circumference, *OU* oculus uterque (“of both eyes”), *qHS* quaque hora somni (“nightly”), *SMA* spinal muscular atrophy, *VUS* variant of unclear clinical significance, *XLID* x-linked intellectual disability

### Genetic investigations

All individuals remained undiagnosed following standard genetic investigations, including genomic microarray (eight of eight individuals), Fragile X testing (six of eight individuals), and/or ID panel testing (three of eight individuals). Each kindred (proband and parents) next underwent clinical exome trio analysis, followed by distributed case-matching of genetically and phenotypically like patients using GeneMatcher [6]. Interestingly, seven individuals (1–7) were each heterozygous for the specific de novo substitution *TRPM3*(NM_020952.4):c.2509G>A, NP_066003.3:p.(Val837Met). This change is not represented in GnomAD, and is predicted to be damaging (scaled CADD score 25.4; SIFT score 0.000; PolyPhen-2 score 0.998) [7–10]. The ACMG categorization of this variant is “pathogenic” on the basis of criteria PS2, PS4, PM1, PM2, and PP3 [11]. The eighth (final) individual was heterozygous for a private substitution, c.2810C>A, p.(Pro937Gln), observed once in GnomAD (allele frequency: 3.98 × 10^−6^) and predicted to be damaging (scaled CADD score 28.8; SIFT score 0.000; PolyPhen-2 score 1.000). This variant meets ACMG criterion PS2, and is categorized as a variant of unknown significance. Of note, review of the other de novo coding variants in individual 8 further demonstrated a heterozygous splice-acceptor site deletion in the DNA damage-response protein *DDB1* [(NM_001923.4):c.550-4_554delCCAGGACCC]. Although *DDB1* variants are not, as far as we are aware, directly implicated in any human disease, the *TRPM3* variant in individual 8 remains an unclassified variant pending additional reports.

**Fig. 2:**
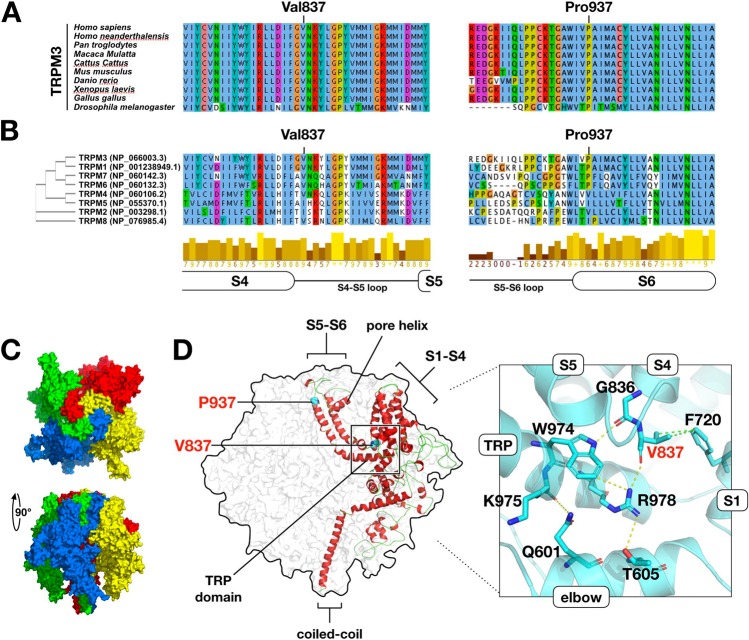
Predicted effects of TRPM3 substitutions. **a** Val837 and Pro937 are highly conserved. **b** Val837 and Pro937 are conserved across multiple TRP(melastatin) subfamily paralogues. **c** Structural model of residues 340–1098 of TRPM3 (NP_066003.3), based on TRPM7 (PDB:6bwd) (ref. [13]). The modeled portion of TRPM3 is 63% identical to the corresponding TRPM7 peptide sequence. Four monomers are radially arranged around a central channel pore. Distinct structural domains are formed by transmembrane helices S1–S4 (voltage-sensing domain), and helices S5–S6 (pore-forming domain). The selectivity filter is formed by a short “pore helix” situated in the S5–S6 loop. The TRP domain, a horizontal alpha-helix at the position indicated, is proposed to couple the movements of the voltage-sensing and pore-forming domains (ref. [13]). **d** Overview of the model showing positions of substituted positions Val837 and Pro937. Val837 resides in the S4, S5 linker region, where it is predicted to form a hydrogen bond with Arg978 (TRP domain) and two Van der Waals contacts with Phe720 (helix S1). Arg978 is essential for channel function in TRPM6, and is proposed to make additional intra- and inter-helical contacts as shown (ref. [15]). The Met837-substituted model (not shown) adopts a similar conformation but is capable of making only a single Van der Waals contact with Phe720. Pro937 is situated in the pore-forming domain, at the transition point between helix S5 and the pore-forming S5–S6 loop. In the Gln937-substituted model (not shown), helix S6 extends two residues (one half-turn) further into the S5–S6 pore-forming loop

Public databases confirm that heterozygous loss-of-function variants of *TRPM3* are observed in the general population. For instance, heterozygous gnomAD truncating variants occur in 18 of 25 canonical coding exons, and truncating variants are nondepleted as a proportion of all coding variants (ExAC pLI statistic = 0.00%) [7]. Moreover, in DGV [12], copy-loss CNVs intersect multiple constitutive coding exons of *TRPM3*. In contrast, the gnomAD missense variation constraint metric for *TRPM3* (*Z* = +3.18) suggests relative intolerance for in-frame substitution. Because (i) *TRPM3* variants were nonrandomly distributed in our cohort and (ii) functional hemizygosity of *TRPM3* appears tolerated in general-population controls, we reasoned that simple haploinsufficiency was unlikely to be the mechanism of disease in our cohort. To predict the functional consequences of the variants in our patients, we modeled TRPM3 on the recently determined structure [13] of TRPM7 (Fig. 2). Like other TRP channels, TRPM7 is a radial tetramer with spatially distinct voltage-sensing and pore-forming domains (encoded by helices S1–S4 and S5, S6, respectively). Between voltage-sensing and pore-forming domains resides the TRP domain, a conserved “switch” region [13]. The model of TRPM3 suggests at least four hypotheses regarding the functional consequences of a valine-to-methionine substitution at position 837. Firstly, Val837 occupies a crucial position in TRPM3’s S4–S5 linker, a conserved helix which interacts with the TRP domain during gating [13, 14]. In TRPM3, a hydrogen bond is predicted between Val837 and Arg978 of the TRP domain; in TRPM7, the analogous bond (Val982-Arg1115) is essential and even conservative substitutions (e.g., p.Arg1115Gln) render the channel inactive [13, 15]. Secondly, TRPM3 is unique among TRP proteins in that its voltage-sensing domain contains a second, non-canonical, permeation pathway distinct from the central channel pore [16, 17]. Noncanonical conductance in TRPM3 can be abolished by mutations at any of the helix IV residues Trp827, Arg830, and Asp833, or Gly836, the latter being immediately adjacent to Val837 [17]. Thirdly, TRPM3 is among several TRP proteins responsive to phosphoinositides, and Arg978 is one of three TRP domain residues essential for phosphoinositide responsiveness [15, 18]. Fourthly, the tetrameric structure of TRP channels presents the possibility of structural dominant negativity by direct interaction of mutant and nonmutant subunits.

The case for pathogenicity of the p.Pro937Gln variant, observed only once in our cohort, is less clear. This substitution of a conserved, “helix-breaking” proline at the N-terminal extreme of helix S6 is predicted to extend helix S6 by one half-rotation, shortening and reanchoring the flexible pore-forming S5–S6 loop. This variant is regarded as a variant of unclear clinical significance, pending confirmation in additional probands.

## Discussion

In this report, we present eight individuals with ID, hypotonia, epilepsy (seven individuals), and de novo substitutions of *TRPM3*. The primary manifestations of this disorder are nonspecific, and we anticipate that panel- or exome-based sequencing is likely to be the typical means of diagnosis. Notwithstanding a few prior reports describing *TRPM3* variation in humans, this study is the first to definitively assign a clinical phenotype to this gene in multiple unrelated probands. In 2014, the substitution *TRPM3* (NM_020952):c.195A>G, p.(Ile65Met) was identified in an autosomal dominant glaucoma and cataract kindred linked to 9p13-q21 [19]. Although the *TRPM3* variant did cosegregate with the affected haplotype, the critical region was large (~40 Mb; 114 genes), and causality was not established. Secondly, we are aware of a case report of brothers with Becker muscular dystrophy, autism, and a partial (nine-exon) *TRPM3* deletion; however, the deletion did not cosegregate with disease [20]. Thirdly and finally, we know one prior report of a Kabuki-like syndrome in a single individual with a ~1.3Mbp microdeletion encompassing *TRPM3* and three other genes; however, segregation was not assessed, as parents were unavailable [21]. This report is therefore the first to show a consistent *TRPM3*-related clinical phenotype across multiple unrelated kindreds.

TRPM3 is highly expressed in the brain in humans and other vertebrates [22]. In the developing rat brain, TRPM3 is initially restricted to neurons, but as myelination progresses expression shifts to oligodendrocytes, in which it mediates a calcium current responsive to D-*erythro*-sphingosine (a byproduct of myelin sphingolipid biosynthesis) [23]. The patients in this study did not show differences in cerebral myelination, although a minority of patients did show nonspecific cerebral white matter volume loss.

A well-characterized function of TRPM3 in the literature is its role in thermal nociception. Together with the capsaicin receptor, TRPV1, and the allyl isothiocyanate (wasabi) receptor, TRPA1, TRPM3 is one of three TRP channels required for noxious heat sensation in thermosensory neurons [24]. In mouse, TRPM3 is expressed in sensory neurons from dorsal root and trigeminal ganglia, and *Trpm3*^*−/−*^ mice display attenuated nocifensive behavior after heat or dermal pregnenolone sulfate injection [25]. In this study, abnormal pain perception was endorsed in two individuals on specific questioning, but this feature was not consistent across the entire cohort, nor was it the presenting complaint in any patient. In the future, it may be interesting to objectively assess thermal nociception in *TRPM3* patients by means of contact heat-evoked potentials, an electrophysiological technique requiring specific apparatus unavailable for use in this report.

This report is congruent with that of Hamdan et al. [2], who find that many of the identifiable variants in patients with DEE are recurrent, frame-preserving, de novo substitutions in channels or receptors expressed at the neuronal plasma membrane. Our findings suggest that *TRPM3* is a locus for ID and epilepsy, and should be included in genetic panels targeting these indications.
